# Chrono-optimizing vaccine administration: a systematic review and meta-analysis

**DOI:** 10.3389/fpubh.2025.1516523

**Published:** 2025-04-07

**Authors:** Koen Vink, Johannes Kusters, Jacco Wallinga

**Affiliations:** ^1^Centre for Infectious Disease Control, National Institute for Public Health and the Environment, Bilthoven, Netherlands; ^2^Department of Epidemiology and Data Science, Amsterdam University Medical Centre, Amsterdam, Netherlands; ^3^Department of Biomedical Data Sciences, Leiden University Medical Centre, Leiden, Netherlands

**Keywords:** influenza, COVID-19, vaccination timing, vaccine response, vaccine effectiveness, chronobiology

## Abstract

**Background:**

Increasing evidence suggests that vaccine responses may vary based on the time of day of administration. This systematic review provides a comprehensive overview of the impact of vaccination timing on immune responses, to assess its potential role in optimizing vaccination programs.

**Methods:**

A systematic literature search was performed in Embase, Medline and Scopus to identify eligible observational studies and clinical trials that assessed immune responses following vaccination at different times of the day in humans. A meta-analysis of clinical trials was conducted to quantify the effect size of vaccination timing on antibody responses.

**Results:**

The search identified 17 studies that compared vaccine responses at different times of the day, covering vaccinations against COVID-19 (9), influenza (5), hepatitis B (2), hepatitis A (1), and pneumococcal infection (1). Eleven out of these 17 studies demonstrated statistically significant effects of vaccination timing on the antibody response, with 10 reporting stronger antibody responses following morning compared to afternoon vaccination. Of the six subgroups with an average age of 60 years and older, five showed significantly stronger antibody responses following morning vaccination, while the sixth showed a significant effect only in men. In contrast, only five out of 16 subgroups with an average age younger than 60 years showed a statistically significant effect of vaccination timing on antibody titers. Similarly, the meta-analysis indicated that receiving influenza vaccination in the morning elicited a stronger antibody response than in the afternoon (SMD = 0.24, 95% CI = 0.01–0.47), with subgroup analyses revealing a larger effect in adults aged 65 and older (SMD = 0.32, 95% CI = 0.21–0.43) compared to those aged 60 or younger (SMD = 0.00, 95% CI = −0.17–0.17).

**Conclusion:**

Morning vaccination enhanced antibody responses in adults aged 60 years and older, a key demographic for influenza and COVID-19 vaccination. Chrono-optimizing vaccine administration may offer a low-risk, low-cost strategy to boost vaccine effectiveness in this age group.

**Systematic review registration:**

https://inplasy.com/inplasy-2025-1-0060/.

## 1 Introduction

Enhancing vaccine-induced protection against prevalent infections, particularly those associated with severe disease, is essential for reducing the overall burden of infectious diseases. Conventional strategies to enhance vaccine immunogenicity, including the optimization of antigen presentation and the incorporation of novel adjuvants, encounter challenges related to time-consuming safety testing and the potential for adverse events (1). Consequently, low-risk strategies are being explored for their impact on vaccine responses, including physical exercise, sufficient sleep, and the timing of vaccine administration (2–5).

The time of day at which vaccines are administered may influence vaccine responses due to daily fluctuations in various components of the immune system. These circadian rhythms have been described in cytokine responses (6, 7), circulating leukocyte counts (6, 8), sensitivity to pathogen-associated molecular patterns (7, 8), and the activity of both innate and adaptive immune cells (7, 9, 10). These rhythms are controlled by cell-intrinsic circadian clocks, composed of so-called *CLOCK* proteins, that regulate 24-h cycles in cellular functions by coordinating transcriptional and translational feedback loops (11).

Aligning the time of vaccine administration with these oscillations in the immune system could enhance immune responses and potentially increase vaccine effectiveness with minimal risk (12, 13). This raises the question whether there is an optimal time for vaccine administration to maximize immunogenicity. Recently a substantial number of observational studies and randomized clinical trials (RCTs) have appeared on this topic, and a synthesis of the currently available evidence is an essential step in establishing whether there is such an optimal vaccination time.

This systematic review aims to provide a comprehensive overview of the current evidence from observational and experimental studies that compare vaccine responses based on the timing of administration throughout the day. The objective is to assess the overall impact of vaccination timing on immune responses and identify key areas for further research to better understand how vaccine administration timing affects immunogenicity and its potential role in optimizing vaccination programs.

## 2 Methods

This study followed the Preferred Reporting Items for Systematic Reviews and Meta-Analyses (PRISMA) guidelines (Supplementary Table S1) (14). The protocol of this systematic review was registered on INPLASY (registration number: INPLASY202510060).

### 2.1 literature search and study selection

A systematic literature search was conducted in the Embase, Medline and Scopus databases to identify eligible studies for this review, covering all records up to January 31, 2025. The detailed search query is provided in the Supplementary Table S2. Two researchers independently screened the identified studies. After removing duplicates, the titles and abstracts of the remaining records were screened for eligibility. Studies qualified for inclusion if they measured antigen-specific antibody or T-cell responses following vaccination, and if these immune responses were compared between participants vaccinated at different time points during the day. Studies were excluded if they did not provide sufficient data on the timing of vaccination or involved non-human subjects. A full-text review was conducted to confirm whether each study met the eligibility criteria. In cases of disagreement, a third researcher was consulted to reach a consensus. In addition to the database search, a reference list check of the included studies was conducted to ensure comprehensiveness.

Included studies were classified as RCTs if the time of vaccine administration was randomized; otherwise, they were categorized as observational studies. Observational studies, in contrast to RCTs, are more prone to confounding factors and other sources of bias that may influence immune outcomes. Consequently, their findings were interpreted with greater caution due to their inherent susceptibility to bias and lower level of evidence for establishing causality. A clear distinction between these two study designs was maintained throughout this review.

A meta-analysis approach was employed, including only RCTs, to estimate the overall effect size of vaccination timing on antibody responses. RCTs were eligible if antibody titers were measured at least 1 month post-vaccination, the study population did not consist of immunocompromised patients, and data were available for the analysis.

### 2.2 Risk of bias assessment

The risk of bias of the included studies was critically appraised by two assessors using Cochrane's Risk of Bias tool 2 (RoB2) for (cluster-)RCTs (15), and Risk Of Bias In Non-randomized Studies of Interventions tool (ROBINS-I) for observational studies (16). A final consensus judgement was reached for each study by considering the evaluations of both assessors, and if necessary a third assessor was consulted.

Publication bias was assessed by checking clinical trial registers for ongoing or unpublished studies.

### 2.3 Data extraction and organization

Data on study design, location, number of subjects and their characteristics, vaccination type, vaccination time, and study outcomes were extracted from the included studies to assess the presence and direction of any effect of vaccination timing. Additional data required for quantifying the overall effect size of vaccination timing on antibody responses were obtained from published supplementary materials of eligible RCTs and through contacting the authors. These data included the mean and standard deviation (SD) of antigen-specific antibody titers measured 1 month post-vaccination. All reported titers were log-transformed for standardization. These log-transformed antibody titers will be referred to as simply “antibody titers”.

### 2.4 Outcomes

The primary outcome of the meta-analysis was the standardized mean difference (SMD) in antibody titers 1 month post-vaccination between morning and afternoon vaccine administrations. Secondary outcomes included the potential modifying effects of age and sex on the relationship between vaccination timing and the antibody response. Group sizes, along with the mean and SD of antibody titers were used to calculate the SMD in titer levels between morning and afternoon vaccination, as well as the corresponding variance and standard error.

### 2.5 Statistical meta-analysis

A three-level random-effects model was used to obtain a pooled effect estimate with confidence intervals for the difference in the antibody response between morning and afternoon vaccination. This model corrected for the correlation between the multiple effect sizes within each study. Heterogeneity between the selected studies was assessed using the *tau*^2^, Cochran's *Q* and *I*^2^statistics. Subgroup analyses were conducted based on sex, age group, and vaccine strain to explore potential sources of heterogeneity and assess whether these variables moderated the relationship between vaccination timing and the antibody response. These subgroups were pre-specified based on prior evidence suggesting that these variables could influence vaccine responses (3). All analyses were performed in R (version 4.3.0, R Core Team, Vienna, Austria) with the “*metafor*” (17) package.

## 3 Results

### 3.1 Systematic review

#### 3.1.1 Search outcome and general characteristics of studies

**Figure 1:**
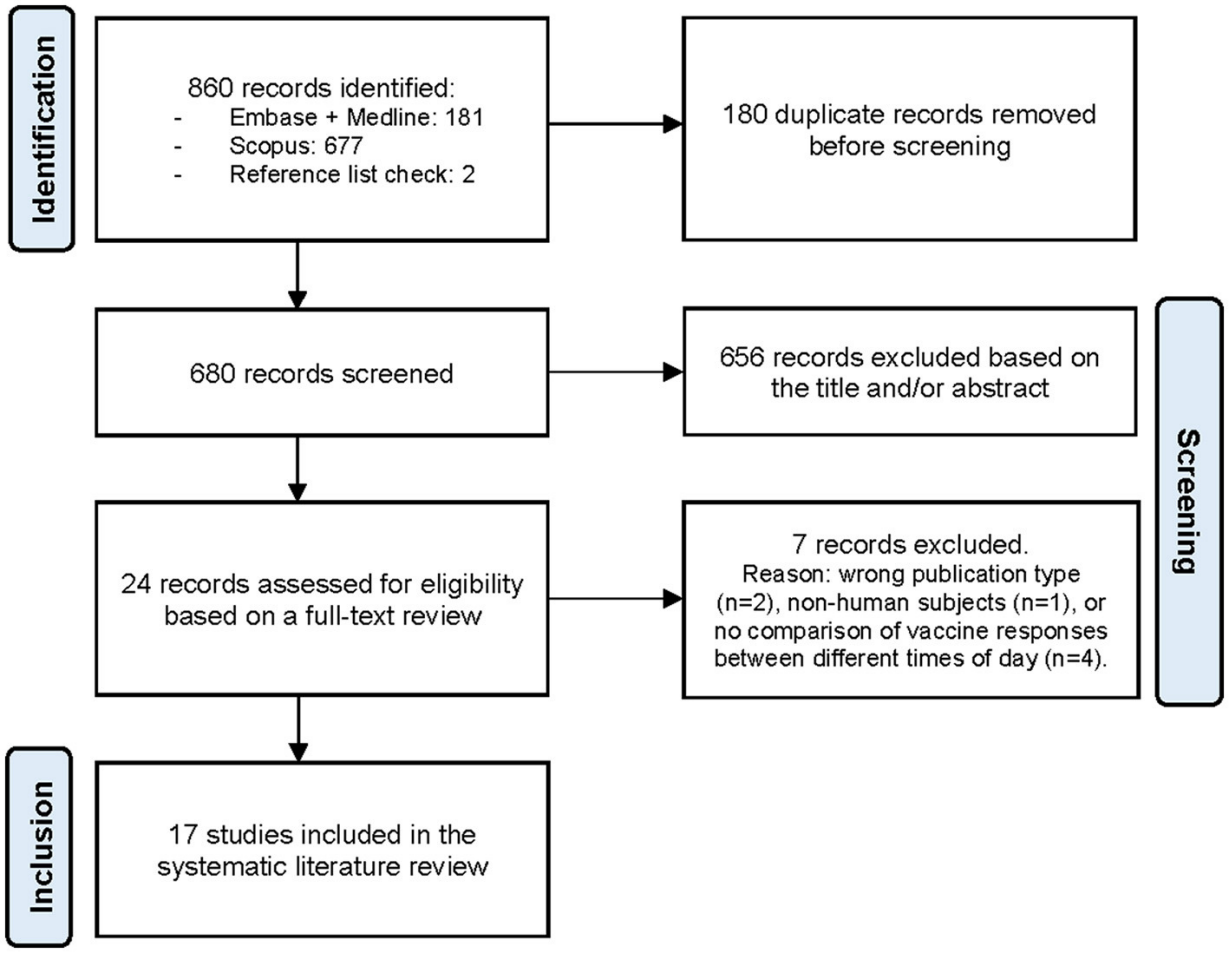
The PRISMA (Preferred Reporting Items for Systematic Reviews and Meta-Analyses) flow diagram illustrating the study selection process (14).

A total of 860 records were identified through the literature search, of which 17 met the eligibility criteria and were included in the systematic review to assess the presence of an effect of vaccination timing on immune responses (Figure 1) (18–34). Among these, 13 were observational studies (20–22, 24–30, 32–34) and four were RCTs (18, 19, 23, 31). The studies were categorized by the type of vaccine administered: (I) influenza vaccination, (II) SARS-CoV-2 vaccination, and (III) vaccination targeting other infectious diseases.

#### 3.1.2 Influenza vaccination

Five out of the 17 included studies investigated the effect of vaccination timing on immune responses to influenza vaccines (18–22). Participants in these studies received standard-practice trivalent or quadrivalent inactivated influenza vaccines either in the morning or afternoon. Antibody responses were assessed for multiple vaccine strains, i.e., A/H1N1, A/H3N2 and B strain influenza. The characteristics and findings of these studies are summarized in Table 1.

**Table 1:** Characteristics and findings of studies investigating the effect of vaccination timing: **influenza** vaccination.

	**Author (year)**	**Location**	**Vaccination time**	**Titer meas. (week)**	**Study size (n)**	**Sex (♀)**	**Age (years)**	**Influenza vaccine strain**	**Stronger antibody response**				**Remarks**
									**AM**	**NS**	**PM**	**Sign**.	
Randomized controlled trials	Long et al. (2016) (18)	West Midlands, United Kingdom	AM: 9–11 am PM 3–5 pm	0, 4	276	49.3%	≥65; Mean: ~71	A/H1N1	X			*p =* 0.03	
									A/H3N2		X		*p =* 0.35	
									B strain	X			*p =* 0.01	
		Liu et al. (2022) (19)	Guangzhou, China	AM: 9–11 am PM: 3–5 pm	0, 4	389	62.5%	65–75; Mean: 69.2	A/H1N1	X			*p =* 0.05	Predominantly women aged between 65 and 75 showed significantly higher titers following morning vaccination.
									A/H3N2	X			*p =* 0.02	
									B strain		X		*p =* 0.10	
								50–60; Mean: 56.3	A/H1N1		X		*p =* 1.00	
									A/H3N2		X		*p =* 0.99	
								B strain		X		*p =* 0.50	
Observational studies	Langlois et al. (1995) (21)	Houston, United States	8:30 am−5 pm	0, 3–5	707	± 50%	30–60; Mean: 43.9 (±0.9)	A/H1N1				N/Appl	The study only tested for an effect of vaccination timing on the antibody response; no effect observed.
									A/H3N2				N/Appl	
									B strain				N/Appl	
		Langlois et al. (1995) (21)	Princeton, United States	8:30 am−5 pm	0, 3–4	98	± 50%	Mean: 45 (±14.6)	A/H1N1				N/Appl	Significant effect of vaccination timing observed for A/H3N2 (*p < * 0.02); highest titer increase between 11 am and 1 pm.
									A/H3N2				N/Appl	
									B strain				N/Appl	
		Phillips et al. (2008) (20)	Birmingham, United Kingdom	AM: 8–11 am PM: 1–4 pm	0, 4	89	57.3%	≥65; Mean: 73.1 (±5.5)	A/H1N1		X		N/A	Only men vaccinated in the morning elicited a significantly stronger antibody response to the A/H3N2 strain (*p =* 0.03).
									A/H3N2		X		N/A	
									B strain		X		N/A	
		Kurupati et al. (2017) (22)	North Carolina, United States	Before (AM) and after noon (PM)	0, 1, 2–3	139	67%	≥65; Mean: ~77	A/H1N1	X			*p < * 0.05	According to the authors, VNA titer increase was affected by the timing of blood sampling rather than vaccination.
									A/H3N2		X		N/A	
								30–40; Mean: ~34	A/H1N1		X		N/A	
								A/H3N2		X		N/A	

The participants of all studies were vaccinated with standard-practice trivalent (or quadrivalent) inactivated influenza vaccines. AM, morning vaccination; NS, not significant (p > 0.05); PM, afternoon vaccination; N/A, not available; N/Appl, not applicable; and VNA, virus neutralizing antibody; ♀, female.

Evidence supporting a causal relationship between the time of day of influenza vaccination and the strength of antibody responses comes from two RCTs, Long et al. (18) and Liu et al. (19), which demonstrated that morning vaccination (9–11 am) induced stronger antibody responses than afternoon vaccination (3–5 pm) in adults aged 65 years or older (Table 1) (18, 19). However, findings from observational studies were more variable. One study observed that men (aged ≥65 years) vaccinated in the morning had significantly higher anti-A/H3N2 titers, while women exhibited stronger responses following afternoon vaccination, although this lacked statistical significance (Table 1) (20). This does not align with the findings of the two RCTs, which either reported no significant difference between men and women (18), or, through subgroup analyses, revealed that morning vaccination resulted in stronger antibody responses primarily in women (19). Furthermore, Langlois et al. (21) found no association between vaccination timing and antibody responses in the Houston study, but observed significant variations in the 3–4 week increase in anti-A/H3N2 titers in the Princeton study, with the highest increase in those vaccinated between 11 am and 1 pm and the lowest in those vaccinated around 8:30 am and 5 pm (Table 1 and Supplementary Figure S3) (21). These findings highlight the importance of investigating a wide range of vaccination time intervals, rather than just a morning-vs.-afternoon comparison, to better understand the full spectrum of this time-of-day effect.

Several biological and methodological factors may influence the observed impact of influenza vaccination on immune responses. Long et al. (18) found that the benefit of morning vaccination on antibody titers remained consistent across three influenza seasons, despite annual variations in vaccine strains, suggesting that strain differences do not modulate the observed effect (18). However, Langlois et al. (21) found no significant association between vaccination timing and antibody responses after revaccination in the following year, suggesting that prior influenza vaccination may mitigate the time-of-day effect (21). Furthermore, while Kurupati et al. (22) reported increased anti-A/H1N1 responses following morning vaccination in adults aged ≥65 years, they attributed these observations to variations in the timing of blood sampling rather than the timing of vaccine administration (Table 1) (22). This presents a potential methodological issue if the timing of blood sampling is not standardized across participants or adjusted for in the analysis (22).

These findings indicate that while morning administration of the influenza vaccine may enhance antibody responses in older adults, variability in observational studies underscores the need to account for confounding factors such as the time of blood sampling and vaccination history when assessing the impact of vaccination timing on immune responses.

#### 3.1.3 SARS-CoV-2 vaccination

Nine recent studies have explored how time of day influences immune responses to SARS-CoV-2 vaccines, including inactivated (CoronaVac and BBIBP-CorV) (23, 27), mRNA (BNT162b2 and mRNA-1273) (24–26, 29, 30), and adenoviral (AZD1222) vaccine platforms (28, 30, 34). The findings and characteristics of these studies are displayed in Table 2.

**Table 2:** Characteristics and findings of studies investigating the effect of vaccination timing: **SARS-CoV-2** vaccination.

	**Author (year)**	**Location**	**Vaccination time**	**Vaccine type: doses**	**Titer meas. (week)**	**Study size (n)**	**Sex (♀)**	**Age (years)**	**Stronger antibody response**				**Remarks**
									**AM**	**NS**	**PM**	**Sign**.	
Randomized controlled trials	Lai et al. (2023) (23)	Guangzhou, China	AM: 9–11 am PM: 3–5 pm	CoronaVac (Sinovac): 2 doses	0, 8	469	67.8%	18–60; Mean: 33 (±9.3)		X		*p =* 0.873	Second titer measurement was performed on samples collected 4 weeks after the second dose (week 8).
Observational studies	Zhang et al. (2021) (27)	Guangzhou, China	AM: 9–11 am PM: 3–5 pm	BBIBP-CorV (Sinopharm): 2 doses	0, 2, 3, 4, 8	63	58.7%	Median: 26 (IQR = 24, 28)	X			*p < * 0.001	*p < * 0.001 at week 4 and 8. Morning vaccination also resulted in a stronger B and Tfh cell response.
	Wang et al. (2022) (30)	Oxford, United Kingdom	AM: 7–11 am PM: 11 am−10 pm	BNT162b2 (Pfizer) or AZD1222 (AstraZeneca): 1 dose	2–10	2,784	82.7%	16–74			X	*p =* 0.013	78.7%, 19.7%, and 1.6% of the participants contributed 1, 2, and ≥3 samples, respectively. 75.4% received BNT162b2.
	Matryba et al. (2022) (24)	Warsaw, Poland	AM: < 11 am PM: > 3 pm	BNT162b2 (Pfizer): 2 doses	~16	404	76.1%	20–29; Mean: 23.3 (±1.8)		X		*p =* 0.808	
	Yamanaka et al. (2022) (25)	Sapporo, Japan	Morning (AM) and afternoon (PM) were not defined	mRNA-1273 (Moderna): 1 dose	2–7	332	55.4%	Range: 20–64		X		N/A	
	Erber et al. (2023) (34)	Vienna, Austria	9 am−4 pm	AZD1222 (AstraZeneca) 1 dose	0, 3	803	60.4%	21–74; Mean: 42 (±12)	X			N/Appl	Time of vaccination was significantly associated with anti-spike IgG levels in a non-linear manner (*p =* 0.036). The highest titers were observed at 9–11 am, the lowest at 12–2 pm, and intermediate levels at 2–3 pm.
	Lin et al. (2023) (28)	Taipei, Taiwan	7 am−12 pm, 12–5 pm, and 5–10 pm	AZD1222 (AstraZeneca): 1 dose	4, 8	201	48%	Mean: 67	X			*p =* 0.003	Participants were hemodialysis patients. Morning vaccination had higher odds for seroconversion after 1 month, and for remaining seropositive 2 months post-vaccination compared to afternoon/evening vaccination (OR: 3.81, 95% CI: 1.59–9.15, and OR: 2.54, 95% CI: 1.15–5.61, respectively).
	Pighi et al. (2024) (26)	Peschiera del Garda, Italy	< 10 am, 10-11:59 am, 12-1:59 pm, 14–3:59 pm, > 4 pm	BNT162b2 (Pfizer): 1 dose	3	249	60.6%	Mean: 44 (±13)		X		N/A	
	Zahradka et al. (2024) (29)	Prague, Czech Republic	7 am−6 pm	BNT162b2 (Pfizer) or mRNA-1273 (Moderna): 2 doses	~7	553	36.0%	Seroconv.: mean: 63 (IQR: 56, 71) Not seroconv.: mean: 67 (IQR: 58, 72)	X			N/Appl	Participants were immunosuppressed KTRs. 97% received BNT162b2. The odds for seroconversion was higher for those vaccinated in the morning; with every hour of delay of the second dose the odds for seroconversion decreased (OR: 0.84, 95% CI: 0.71–0.998).

AM, morning vaccination; NS, not significant (p > 0.05); PM, afternoon/evening vaccination; N/A, not available; N/Appl, not applicable; seroconv., seroconverted; KTR, kidney transplant recipient; and IQR, interquartile range; ♀, female.

The studies on SARS-CoV-2 vaccination exhibited substantial heterogeneity, with differences in participant age, vaccine type, and comorbidities, which may have contributed to inconsistencies in the observed results. Four out of nine studies, including one RCT, found no significant effect of vaccination timing on antibody responses to SARS-CoV-2 vaccines among relatively young participants (Table 2) (23–26). In contrast, four other observational studies reported enhanced antibody responses following morning vaccination in healthcare workers/professionals (27, 34), hemodialysis patients (28), and immunosuppressed kidney transplant recipients (29) (Table 2). Notably, Zhang et al. (27) was the only study to evaluate the impact of vaccination timing on immune parameters beyond antibody responses. Specifically, they observed significantly higher proportions of (CD138+) antibody-secreting cells, T follicular helper cells, and antigen-specific memory B cells following morning compared to afternoon vaccination (27). Only one observational study reported that receiving SARS-CoV-2 vaccination later in the day (11 am−10 pm or after 1 pm) resulted in stronger anti-spike responses compared to vaccination in the morning (7–11 am or before 1 pm) (Table 2) (30).

The discrepancy in findings across these studies likely stems from heterogeneity in study population characteristics (e.g., age and comorbidity) as well as methodological differences (e.g., variations in the type of vaccine platform, the number of vaccine doses, and length of the follow-up period post-vaccination) between the studies (Table 2).

#### 3.1.4 Vaccination targeting other infectious diseases

Four studies examined the diurnal variation in immune responses to vaccines targeting other infectious diseases as displayed in Table 3 (20, 31–33). These studies used various vaccination platforms, including inactivated hepatitis A vaccines (20), subunit hepatitis B vaccines (31, 32), and polysaccharide pneumococcal vaccines (33).

**Table 3:** Characteristics and findings of studies investigating the effect of vaccination timing: **hepatitis A, hepatitis B, and pneumococcal** vaccination.

	**Author (year)**	**Location**	**Vaccination time**	**Vaccine: doses**	**Titer meas. (week)**	**Study size (n)**	**Sex (♀)**	**Age (years)**	**Stronger antibody response**				**Remarks**
									**AM**	**NS**	**PM**	**Sign**.	
Randomized controlled trials	Karabay et al. (2008) (31)	Bolu, Türkiye	AM: 8–8:30 am PM: 5:30–6 pm	Hepatitis B: 3 doses	4 (after the last dose)	63	57.1%	19–23; Mean: 20.5		X		*p >* 0.05	
Observational studies	Phillips et al. (2008) (20)	Birmingham, United Kingdom	AM: 10 am−12 pm PM: 4–6 pm	Hepatitis A: 1 dose	0 and 4	75	54.7%	Mean: 22.9 (±3.9)		X		N/A	This study was partially randomized. Only men vaccinated in the morning elicited a significantly stronger antibody response (*p =* 0.03).
	Whittaker et al. (2022): Study 1 (33)	Birmingham, United Kingdom	AM: 10 am−12 pm PM: 4–6 pm	Pneumococcal vaccine: 1 dose	0, 1, 4, and 18	75	54.7%	Mean: 22.9 (±3.9)		X		*p =* 0.22	There was no effect of vaccination timing on the IgG response averaged across the polysaccharide serotypes (*p =* 0.22) or for individual serotypes.
	Whittaker et al. (2022): Study 2 (33)	Birmingham, United Kingdom	Morning (AM) and afternoon (PM) were not defined	Pneumococcal vaccine: 1 dose	0, 4, and 24	61	70.5%	Mean: 41.4 (±5.3)		X		*p =* 0.10	There was no effect of vaccination timing on the IgG response averaged across the polysaccharide serotypes (*p =* 0.10) or for individual serotypes.
	Coppeta et al. (2023) (32)	Rome, Italy	AM: 9–11 am PM: 2–4 pm	Hepatitis B: 1 booster dose	0 and 4 to 8	294	65.3%	Mean: 21.7 (±1.7)	X			*p < * 0.05	The participants were vaccinated at birth but had unprotective titers at baseline. Morning vaccination resulted in an increased likelihood of developing protective titers (OR: 1.93, 95% CI: 1.047–3.561).

AM, morning vaccination; NS, not significant (p > 0.05); PM, afternoon/evening vaccination; N/A, not available; ♀, female.

Two out of the four studies favored morning over afternoon vaccination (20, 32), while the other two studies, including one RCT, reported no association between vaccination timing and antibody responses (31, 33). Specifically, Phillips et al. (20) found that men receiving hepatitis A vaccination in the morning had significantly stronger antibody responses compared to those vaccinated in the afternoon, while women showed a non-significant trend toward stronger responses following afternoon vaccination (Table 3) (20). Similarly, Coppeta et al. (32) observed that young adults with unprotective baseline titers who received a morning booster dose of the hepatitis B vaccine had a significantly higher response rate compared to those who were vaccinated in the afternoon (Table 3) (32). These findings contrast with the results of an RCT, which found no statistically significant difference in antibody titers 1 month after the final dose of a three-dose hepatitis B vaccine series in a similar age group (31). Additionally, Whittaker et al. (33) observed no effect of the timing of 23-valent polysaccharide pneumococcal vaccine administration on antigen-specific IgG responses, either averaged across the polysaccharide serotypes, or for individual serotypes, including type 1, 3, 6, 9, 14, 19, and 23 (33).

Due to the limited number of studies for each vaccine type, it is not possible to identify a consistent pattern regarding the effect of vaccination timing for any of these vaccines.

#### 3.1.5 Impact of vaccination timing across age groups

**Figure 2:**
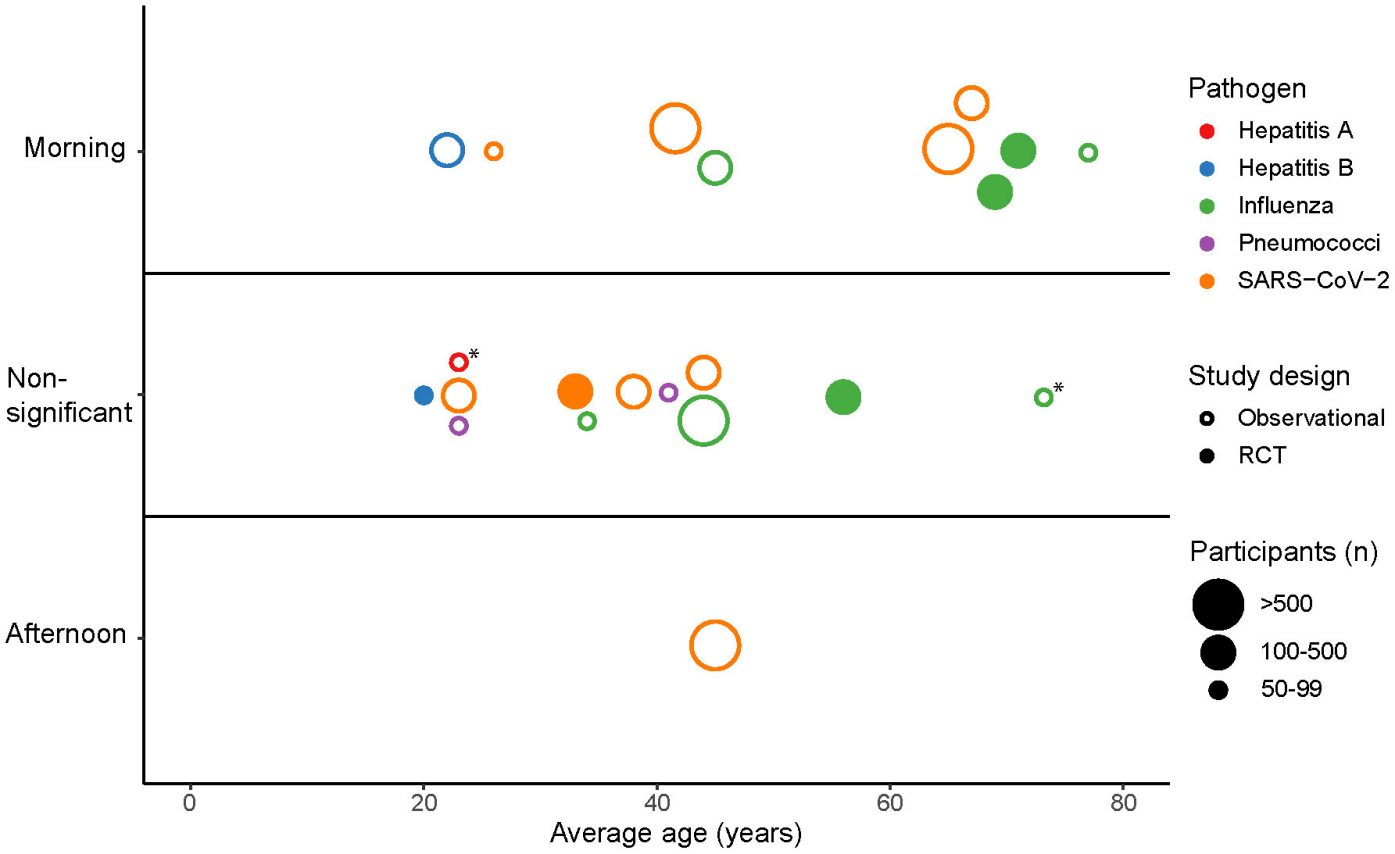
The effect of vaccination timing on antibody responses across all age-based subgroups from the included studies. Points represent subgroups that are categorized as “Morning” or “Afternoon” depending on whether the subgroup showed a significantly stronger antibody response for those vaccinated in the morning or afternoon. Subgroups are categorized as “Non-significant” if no significant difference (*p* > 0.05) in antibody responses was observed between morning and afternoon vaccination. *Men vaccinated in the morning showed a significantly stronger antibody response compared to afternoon vaccination.

The included studies reported age-stratified findings, resulting in a total of 22 subgroups. Figure 2 presents an overview of the effect of vaccination timing on antibody responses across age-based subgroups. In five out of six subgroups with an average age of 60 years or older, morning vaccination induced significantly stronger antibody responses compared to afternoon vaccination, while one subgroup showed this effect only in men. In contrast, only five out of 16 subgroups with an average age younger than 60 years showed a statistically significant effect of vaccination timing on antibody titers. This suggests that the benefits of optimizing the time of day of vaccine administration are more pronounced in older adults.

### 3.2 Risk of bias in the included studies

The results of the risk of bias assessment for the included studies are provided in Supplementary Figure S4. The overall risk of bias was low for most RCTs, but varied across the observational studies. An inherent limitation of observational studies is the potential for confounding, as participants are not randomized and can choose their own preferred vaccination time. Participants with an evening chronotype are therefore less likely to receive vaccinations in the morning, yet none of the studies accounted for chronotype as a potential confounding factor. Several other factors that could influence the effect of vaccination timing on immune responses—such as physical activity (2), sleep (5) and the timing of blood sampling (22)—were also not considered. Furthermore, inconsistencies in follow-up periods across studies may have impacted the interpretation of the effect of vaccination timing on antibody responses, as the duration for which observed differences in antibody titers persist remains unclear.

### 3.3 Meta-analysis: quantification of the effect of vaccination timing on antibody titers

To quantify the effect size of vaccination timing on antibody responses, we employed a meta-analysis approach that included only RCTs to obtain a pooled estimate. Among the three trials with available data, there was high heterogeneity in terms of vaccine type and participant age (18, 19, 23). Therefore, the meta-analysis focussed specifically on the two influenza vaccination trials (Supplementary Figure S5), which reported responses to three different influenza vaccine strains (18, 19).

**Figure 3:**
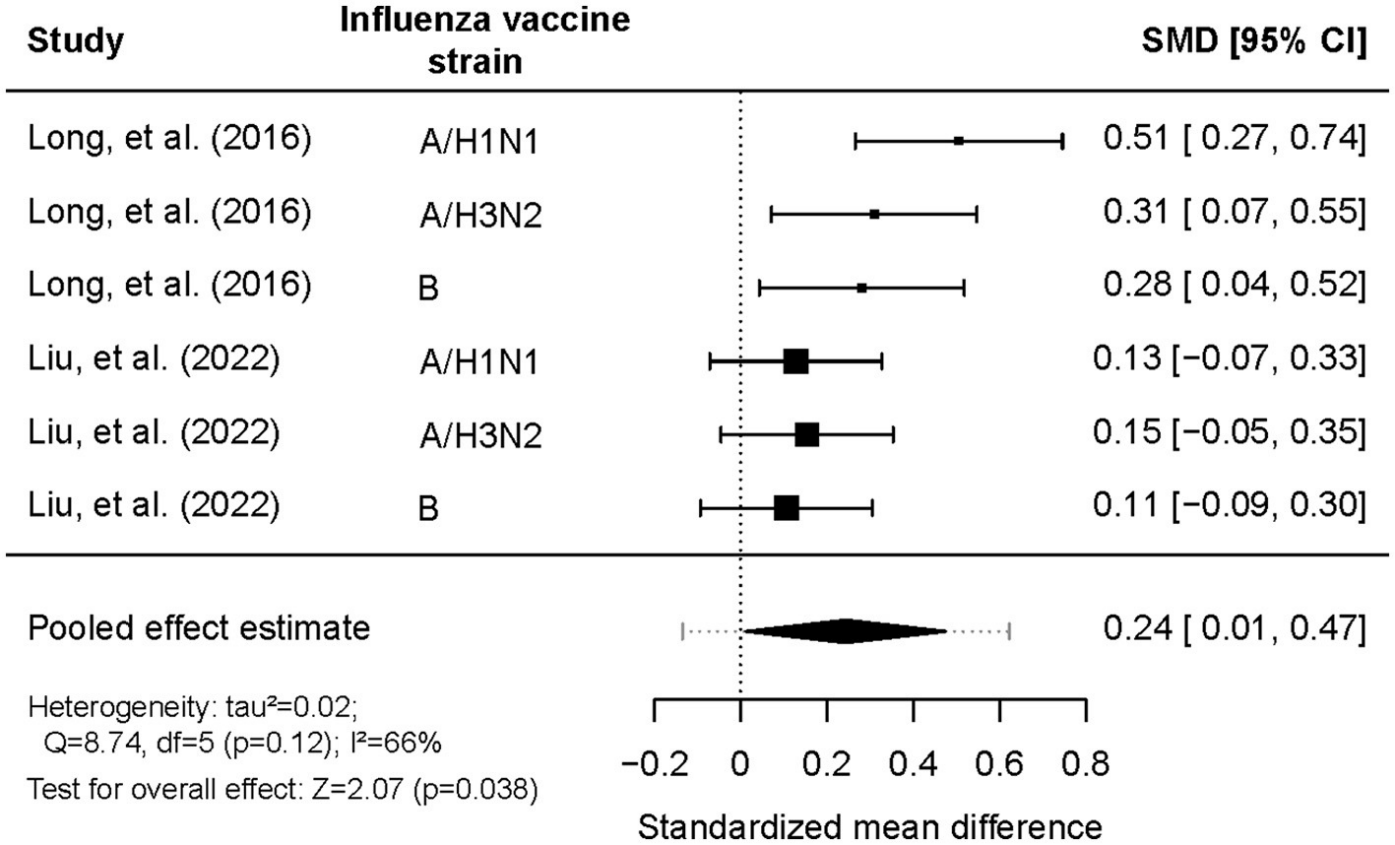
Comparison of log-antibody titers one month post-vaccination between morning and afternoon administration of influenza vaccination.

The SMDs in post-vaccination titers between morning and afternoon vaccination were pooled (Figure 3). All SMDs had positive values, indicating that morning vaccination consistently resulted in higher antibody titers than afternoon vaccination. The pooled SMD was 0.24 (95% CI = 0.01–0.47, *Z* = 2.07, *p* = 0.038), highlighting a statistically significant effect favoring morning vaccination. A substantial level of heterogeneity was detected between the two trials (*tau*^2^ = 0.023; *Q* = 8.74; d*f* = 5; *p* = 0.12; *I*^2^ = 66%). Subgroup analyses revealed that the effect of vaccination timing was significantly stronger among adults aged ≥65 years (SMD = 0.32, 95% CI: 0.21–0.43) than among those aged ≤ 60 years (SMD = 0.00, 95% CI: −0.17–0.17). There were no statistically significant differences between the sexes or the influenza vaccine strains (Table 4). According to the common interpretation of SMD values (with 0.2, 0.5, and 0.8 representing small, medium and large effects, respectively), the effect size among adults aged 65 or older is small to medium.

**Table 4:** Subgroup analysis for the comparison of log-antibody titers between morning and afternoon influenza vaccination.

**Subgroup**	**SMD (95% CI)**	***p*-value**
Age: ≥65 years old	0.32 (0.21–0.43)	< 0.0001
Age: ≤ 60 years old	0.00 (−0.17–0.17)	
Male	0.17 (−0.01–0.42)	0.1669
Female	0.29 (0.04–0.54)	
A/H1N1	0.30 (0.04–0.56)	Ref
A/H3N2	0.24 (−0.03–0.50)	0.5573
B strain	0.20 (−0.07–0.46)	0.3421

SMD, standardized mean difference.

### 3.4 Publication bias

To assess potential publication bias, clinical trial registries were searched for completed but unpublished studies with non-significant findings on the effect of vaccination timing on immune responses. No such studies were identified.

## 4 Discussion

This systematic review identified 17 studies comparing immune responses between morning and afternoon vaccine administration. Eleven out of these 17 studies reported statistically significant effects of vaccination timing, with ten reporting stronger antibody responses following morning vaccination, while one study favored vaccination later in the day. The strongest evidence for diurnal variation was found for influenza vaccines in older adults. Pooled results from two RCTs (18, 19) showed a statistically significant small-to-medium standardized mean difference in antibody titers for adults aged 65 and older, with morning vaccination consistently yielding higher titers 1 month post-vaccination. Mixed results were observed for COVID-19 vaccines, with some studies reporting enhanced immune responses for morning vaccination in specific subgroups, such as hemodialysis and immunocompromised patients (28, 29). The conflicting findings for these studies might be attributed to population characteristics, particularly age, as well as methodological differences between the studies, such as variations in the number of vaccine doses participants received and the length of the follow-up period for blood sampling. Evidence for vaccines targeting other infectious diseases, such as hepatitis and pneumococcal infection was limited and inconsistent.

Although we report statistical evidence supporting a causal relationship between vaccination timing and antibody responses, the underlying mechanisms remain unclear. Circadian rhythms within the immune system arguably play an important role. For instance, both innate and adaptive immune cells peak in peripheral tissues during daytime (the active phase for humans) (8), potentially enhancing immune responses when vaccines are administered at this time. Additionally, circadian variation in cytokine production, antigen presentation and the activity of both innate and adaptive immune cells, as highlighted in previous studies (6–10), may contribute to this time-of-day effect. Further research is needed to explore these diurnal variations in the immune system and to elucidate their influence on vaccine responses.

The age-stratified results reveal a clear trend favoring morning vaccination in adults aged 60 years and older, while this effect was less pronounced in subgroups younger than 60 years. This age-specific effect raises the question why the benefit of morning vaccination is more prominent in older adults. Liu et al. (19) speculated that immunosenescence—the gradual age-related decline in both innate and adaptive immune function—may play a role (19, 35). The overall weaker immune response in older adults might be more affected by circadian oscillations in immune function, making them more sensitive to the timing of vaccination. In contrast, the robust immune function of younger adults may be less susceptible to these circadian rhythms and may obscure any potential benefits of morning vaccination, which might explain the trend toward a non-significant time-of-day effect in this age group (Figure 2). A similar explanation could apply to individuals with impaired immune responses, such as hemodialysis patients (28) and immunosuppressed kidney transplant recipients (29), whose weakened immune system may also be more susceptible to circadian rhythms, thereby amplifying the effects of vaccination timing.

The available studies suggest that the effect of vaccination timing holds for various vaccine platforms (Supplementary Figure S6). The polysaccharide vaccine was the only vaccine platform for which no effect of vaccination timing was detected. Whittaker et al. (33) suggest that this might be because polysaccharide vaccines trigger a thymus-independent response (33). These vaccines primarily stimulate B cells directly, without T cell help, as this requires peptide presentation by antigen-presenting cells (36). T cell functions, including differentiation, activation, and migration, are strongly influenced by circadian cues (10, 37). Although B cells also show circadian variation, such as in *CLOCK* gene expression and circulating numbers, these rhythms may be less directly tied to functional changes compared to T cells (10, 33, 37). Therefore, the effect of vaccination timing might be more pronounced for thymus-dependent vaccines.

The majority of the reviewed studies did not report significant differences between men and women in the effect of vaccination timing on immune responses. However, stratified analyses of two studies reported conflicting results: one found a significant time-of-day effect exclusively in women (19), while the other observed this effect only in men (20). It is well established that vaccine responses differ between the sexes. Women generally exhibit stronger humoral and cellular responses to vaccines than men, potentially due to differences in immunoregulatory hormones, like estrogens and androgens (38). However, there is limited evidence on sex-based differences in circadian rhythms of the immune system that supports a different optimal vaccination time for men and women (39).

Potential confounding factors affecting the effect of vaccination timing on immune responses include the timing of blood sampling (22), vaccination history (21) and lifestyle factors, such as sleep quality and chronotype. Sufficient sleep has been shown to enhance immune responses, whereas sleep deprivation and chronic insomnia are risk factors for impaired vaccine responses, as observed with influenza (40–42) and hepatitis A vaccines (43, 44). Nightshift work, which leads to circadian misalignment of the biological clock, has similarly been associated with reduced vaccine effectiveness (45, 46). Furthermore, the optimal time for vaccination might vary between individuals with different chronotypes, as they exhibit inherent variations in their circadian phase (47). Considering an individual's circadian phase, rather than the time of day, might provide a more accurate prediction of the optimal time for vaccine administration. Future studies investigating the effect of vaccination timing should take these factors into account.

An important public health question is whether the enhanced antibody responses observed following morning vaccination translate to a meaningful increase in vaccine effectiveness. Although higher antibody titers typically correlate with better immune protection, they do not necessarily translate to an increase in vaccine effectiveness. A large cohort study by Hazan et al. (12), analyzing timestamped COVID-19 vaccination data from over 1.3 million individuals, showed that morning and early afternoon vaccination was associated with significantly lower rates of breakthrough infections compared to evening vaccination (12). This finding was consistent across both the standard 2-dose series and booster doses of BNT162b2. Stratified analyses revealed that this effect was significant only in individuals under 20 and over 50 years of age. The relationship between vaccination timing and infection risk followed a sinusoidal pattern, with lower infection risk observed for morning-to-early-afternoon vaccination, rising to higher risk levels for late-afternoon-to-evening vaccination (12). Based on the peak and trough of this relationship, Hazan et al. estimated that optimizing the time of vaccination might improve vaccine effectiveness by 8.6–25% (12). A similar sinusoidal pattern was observed in a recent cohort study of children younger than 6 years (*n* > 250,000), where varicella vaccination in the morning and afternoon was associated with lower infection rates than evening vaccination (13). These findings suggest that vaccination timing could play an important role in optimizing vaccine effectiveness, warranting further investigation across different vaccines and populations.

An inherent limitation of a systematic review on this topic is the limited number of RCTs. Although two trials provided a preliminary effect size estimate across three influenza vaccine strains, the current data do not suffice for a robust outcome of a meta-analysis. The results of these analyses should therefore be interpreted with caution. Beyond influenza and COVID-19 vaccines, there is a notable lack of research on other vaccine types and limited data from diverse regions, particularly low-income countries. These gaps underscore the need for more RCTs investigating the effect of vaccination timing on immune responses across a broader range of vaccine types and populations. For example, no studies have yet examined how vaccination timing influences immune responses in children, despite the widespread administration of vaccines in this age group. Furthermore, future studies should treat vaccination timing as a continuous variable rather than a binary one (morning vs. afternoon), as this may help pinpoint the optimal time for immunization. Finally, future research should assess how vaccination timing affects long-term antibody responses, memory B cell formation, and T cell activity to better understand its role in sustained immune protection.

## 5 Conclusion

This systematic review on the effect of vaccination timing on immune responses suggests that morning vaccination induces stronger antibody responses compared to afternoon vaccination, particularly in adults over 60 years of age. Notably, a causal relationship has been established between morning vaccination and enhanced antibody responses to influenza vaccination in this age group. Since vaccines against influenza and COVID-19 are widely recommended for adults over 60, these findings potentially hold significant public health implications at population level. Implementing chrono-optimized vaccination strategies into immunization programs could provide a low-risk, low-cost approach to enhance vaccine effectiveness, particularly in older adults who are at higher risk of severe disease. Prioritizing morning vaccination for this age group in clinical settings and general practices could be a feasible and practical strategy to maximize immune responses to routine vaccines, such as influenza and COVID-19 vaccines. Future research should further investigate the effects of vaccination timing on vaccine effectiveness, to assess the potential benefits of chrono-optimizing vaccination programs. To support this, future vaccination trials should systematically record the time of day at which vaccines are administered, providing evidence that public health agencies and policymakers can use to consider incorporating vaccination timing into immunization guidelines.

## Data Availability

The data used in the meta-analysis is subject to the following licenses/restrictions: Data from Liu et al. (19) is publicly available and can be found in their supplementary materials (https://pmc.ncbi.nlm.nih.gov/articles/PMC9574181/). Data from Long et al. (18) is not publicly available and was obtained by contacting the authors. Requests for access to this dataset should be directed to the corresponding author.
