# Supplementary material for: Chrono-optimizing vaccine administration: a systematic review and meta-analysis
Source: Front Public Health. 2025 Apr 7;13:1516523. doi: 10.3389/fpubh.2025.1516523 (PMC12009823; doi:10.3389/fpubh.2025.1516523)
Supplement: Supplementary file 1 [file Data_Sheet_1.docx]

**Supplementary materials – Chrono-optimizing vaccine administration: a systematic review and meta-analysis (*Vink et al.*)**

***Table S1****:* PRISMA 2020 item checklist

| **Section and Topic** | **Item #** | **Checklist item** | **Location where item**  **is reported** |
| --- | --- | --- | --- |
| **TITLE** | | |  |
| Title | 1 | Identify the report as a systematic review. | 1^st^ page |
| **ABSTRACT** | | |  |
| Abstract | 2 | See the PRISMA 2020 for Abstracts checklist. | 1^st^ page |
| **INTRODUCTION** | | |  |
| Rationale | 3 | Describe the rationale for the review in the context of existing knowledge. | Section 1 |
| Objectives | 4 | Provide an explicit statement of the objective(s) or question(s) the review addresses. | Section 1 |
| **METHODS** | | |  |
| Eligibility criteria | 5 | Specify the inclusion and exclusion criteria for the review and how studies were grouped for the syntheses. | Section 2.1 |
| Information sources | 6 | Specify all databases, registers, websites, organisations, reference lists and other sources searched or consulted to identify studies. Specify the date when each source was last searched or consulted. | Section 2.1 |
| Search strategy | 7 | Present the full search strategies for all databases, registers and websites, including any filters and limits used. | Section 2.1 and Supplement S2 |
| Selection process | 8 | Specify the methods used to decide whether a study met the inclusion criteria of the review, including how many reviewers screened each record and each report retrieved, whether they worked independently, and if applicable, details of automation tools used in the process. | Section 2.1 |
| Data collection process | 9 | Specify the methods used to collect data from reports, including how many reviewers collected data from each report, whether they worked independently, any processes for obtaining or confirming data from study investigators, and if applicable, details of automation tools used in the process. | Section 2.3 |
| Data items | 10a | List and define all outcomes for which data were sought. Specify whether all results that were compatible with each outcome domain in each study were sought (e.g. for all measures, time points, analyses), and if not, the methods used to decide which results to collect. | Section 2.3 and 2.4 |
|  | 10b | List and define all other variables for which data were sought (e.g. participant and intervention characteristics, funding sources). Describe any assumptions made about any missing or unclear information. | Section 2.3 and 2.4 |
| Study risk of bias assessment | 11 | Specify the methods used to assess risk of bias in the included studies, including details of the tool(s) used, how many reviewers assessed each study and whether they worked independently, and if applicable, details of automation tools used in the process. | Section 2.2 |
| Effect measures | 12 | Specify for each outcome the effect measure(s) (e.g. risk ratio, mean difference) used in the synthesis or presentation of results. | Section 2.4 |
| Synthesis methods | 13a | Describe the processes used to decide which studies were eligible for each synthesis (e.g. tabulating the study intervention characteristics and comparing against the planned groups for each synthesis (item #5)). | Section 2.1 |
|  | 13b | Describe any methods required to prepare the data for presentation or synthesis, such as handling of missing summary statistics, or data conversions. | Section 2.3 |
|  | 13c | Describe any methods used to tabulate or visually display results of individual studies and syntheses. | N/A |
|  | 13d | Describe any methods used to synthesize results and provide a rationale for the choice(s). If meta-analysis was performed, describe the model(s), method(s) to identify the presence and extent of statistical heterogeneity, and software package(s) used. | Section 2.5 |
|  | 13e | Describe any methods used to explore possible causes of heterogeneity among study results (e.g. subgroup analysis, meta-regression). | Section 2.5 |
|  | 13f | Describe any sensitivity analyses conducted to assess robustness of the synthesized results. | N/A |
| Reporting bias assessment | 14 | Describe any methods used to assess risk of bias due to missing results in a synthesis (arising from reporting biases). | Section 2.2 |
| Certainty assessment | 15 | Describe any methods used to assess certainty (or confidence) in the body of evidence for an outcome. | Section 2.5 |
| **RESULTS** | | | |
| Study selection | 16a | Describe the results of the search and selection process, from the number of records identified in the search to the number of studies included in the review, ideally using a flow diagram. | Section 3.1.1 and Figure 1 |
|  | 16b | Cite studies that might appear to meet the inclusion criteria, but which were excluded, and explain why they were excluded. | N/A |
| Study characteristics | 17 | Cite each included study and present its characteristics. | Table 1, 2 and 3 |
| Risk of bias in studies | 18 | Present assessments of risk of bias for each included study. | Section 3.2 and Figure S4 |
| Results of individual studies | 19 | For all outcomes, present, for each study: (a) summary statistics for each group (where appropriate) and (b) an effect estimate and its precision (e.g. confidence/credible interval), ideally using structured tables or plots. | Section 3.3, Table 1, 2 and 3, and Figure 2 and 3 |
| Results of syntheses | 20a | For each synthesis, briefly summarise the characteristics and risk of bias among contributing studies. | Section 3.2 and 3.4 |
|  | 20b | Present results of all statistical syntheses conducted. If meta-analysis was done, present for each the summary estimate and its precision (e.g. confidence/credible interval) and measures of statistical heterogeneity. If comparing groups, describe the direction of the effect. | Section 3.3, Figure 3 and Table S6 |
|  | 20c | Present results of all investigations of possible causes of heterogeneity among study results. | Section 3.3, Figure 3 and Table S6. |
|  | 20d | Present results of all sensitivity analyses conducted to assess the robustness of the synthesized results. | N/A |
| Reporting biases | 21 | Present assessments of risk of bias due to missing results (arising from reporting biases) for each synthesis assessed. | Section 3.4 |
| Certainty of evidence | 22 | Present assessments of certainty (or confidence) in the body of evidence for each outcome assessed. | Section 3.3 |
| **DISCUSSION** | | | |
| Discussion | 23a | Provide a general interpretation of the results in the context of other evidence. | Section 4 |
|  | 23b | Discuss any limitations of the evidence included in the review. | Section 4 |
|  | 23c | Discuss any limitations of the review processes used. | Section 4 |
|  | 23d | Discuss implications of the results for practice, policy, and future research. | Section 4 and 5 |
| **OTHER INFORMATION** | | | |
| Registration and protocol | 24a | Provide registration information for the review, including register name and registration number, or state that the review was not registered. | Section 2 (first paragraph) |
|  | 24b | Indicate where the review protocol can be accessed, or state that a protocol was not prepared. | Section 2 (first paragraph) |
|  | 24c | Describe and explain any amendments to information provided at registration or in the protocol. | N/A |
| Support | 25 | Describe sources of financial or non-financial support for the review, and the role of the funders or sponsors in the review. | Section 11 |
| Competing interests | 26 | Declare any competing interests of review authors. | Section 6 |

**S2: Systematic literature search in Embase, Medline and Scopus**

**Table S2.1:** Search string and results from the **Embase** and **Medline** databases

| *No.* | *Query* | *Results (n)* | *Remarks* |
| --- | --- | --- | --- |
| #1 | 'biological rhythm'/exp/mj OR 'biological rhythm*':ti OR (('vaccin*' NEAR/3 'tim*'):ti) | 74798 |  |
| #2 | 'circadian rhythm'/exp OR 'circadian*':ti OR 'diurnal variat*':ti OR 'diurnal rhythm*':ti OR 'chronovaccinat*':ti | 111451 |  |
| #3 | 'immune response'/exp/mj OR 'immun* respons*':ti OR 'humoral immunity'/exp/mj OR 'humoral immun*':ti OR 'antibody titer'/exp/mj OR 'antibody response'/exp OR 'antibod* respons*':ti OR 'antibod* product*':ti OR 'antibody titer*':ti OR 'vaccin* effectiv*':ti OR 'vaccin* efficac*':ti OR 'cellular immunity'/exp/mj OR 'cellular immunity':ti OR 't lymphocyt*':ti OR 't cell*':ti | 562221 |  |
| #4 | 'vaccine'/exp/mj OR 'vaccination'/exp/mj OR 'vaccin*':ti | 365334 |  |
| #5 | 'influenza vaccine'/exp/mj OR 'influenza vaccination'/exp/mj OR 'influenza vaccin*':ti | 29484 |  |
| #6 | 'sars-cov-2 vaccine'/exp/mj OR 'covid 19 vaccination'/exp/mj OR 'covid 19 vaccin*':ti OR 'covid* vaccin*':ti OR 'corona* vaccin*':ti | 37629 |  |
| #7 | (#1 OR #2) AND #3 AND #4 | 181 |  |
| #8 | (#1 OR #2) AND #3 AND #5 | 41 | Influenza vaccination |
| #9 | (#1 OR #2) AND #3 AND #6 | 40 | SARS-CoV2 vaccination |
| #10 | #7 NOT (#8 OR #9) | 100 | Other types of vaccines |
| #11 | 'biological rhythm'/exp OR 'biological rhythm*':ti,ab OR (('vaccin*' NEAR/3 'tim*'):ti,ab) | 223568 |  |
| #12 | 'circadian rhythm'/exp OR 'circadian*':ti,ab OR 'diurnal variat*':ti,ab OR 'diurnal rhythm*':ti,ab OR 'chronovaccinat*':ti,ab | 130557 |  |
| #13 | 'immune response'/exp OR 'immun* respons*':ti,ab OR 'humoral immunity'/exp OR 'humoral immun*':ti,ab OR 'antibody titer'/exp OR 'antibody response'/exp OR 'antibod* respons*':ti,ab OR 'antibod* product*':ti,ab OR 'antibody titer*':ti,ab OR 'vaccin* effectiv*':ti,ab OR 'vaccin* efficac*':ti,ab OR 'cellular immunity'/exp OR 'cellular immunity':ti,ab OR 't lymphocyt*':ti,ab OR 't cell*':ti,ab | 1732931 |  |
| #14 | 'vaccine'/exp OR 'vaccination'/exp OR 'vaccin*':ti,ab | 690606 |  |
| #15 | 'influenza vaccine'/exp OR 'influenza vaccination'/exp OR 'influenza vaccin*':ti,ab | 57026 |  |
| #16 | 'sars-cov-2 vaccine'/exp OR 'covid 19 vaccination'/exp OR 'covid 19 vaccin*':ti,ab OR 'covid* vaccin*':ti,ab OR 'corona* vaccin*':ti,ab | 59282 |  |
| #17 | (#11 OR #12) AND #13 AND #14 | 5516 |  |
| #18 | (#11 OR #12) AND #13 AND #15 | 906 | Influenza vaccination |
| #19 | (#11 OR #12) AND #13 AND #16 | 1305 | SARS-CoV2 vaccination |
| #20 | #17 NOT (#18 OR #19) | 3363 | Other types of vaccines |
| #21 | vaccin*':ti AND ('time*':ti OR 'timing*':ti OR 'circadian*':ti OR 'diurnal*':ti) AND ('immun*':ti OR 'antibod*':ti OR 'respon*':ti OR 'titer*':ti OR 'cellular*':ti OR 't cell*':ti OR 'humoral*':ti) | 569 |  |
| #22 | #18 NOT #8 | 865 |  |
| #23 | #19 NOT #9 | 1265 |  |
| #24 | #20 NOT #10 | 3273 |  |
| #25 | #21 AND #22 | 9 |  |
| #26 | #21 AND #23 | 31 |  |
| #27 | #21 AND #24 | 53 |  |
| #28 | #8 OR #25 | 50 |  |
| #29 | #28 AND ('Article'/it OR 'Editorial'/it OR 'Letter'/it OR 'Preprint'/it OR 'Review'/it) | 44 |  |
| #30 | #28 AND ('article'/it OR 'editorial'/it OR 'letter'/it OR 'preprint'/it OR 'review'/it) AND [1990-2024]/py | 43 |  |
| #31 | #28 AND ('article'/it OR 'editorial'/it OR 'letter'/it OR 'preprint'/it OR 'review'/it) AND [1990-2024]/py NOT [animals]/lim | **37** | Final result for influenza-specific records |
| #32 | #9 OR #26 | 71 |  |
| #33 | #32 AND ('Article'/it OR 'Article in Press'/it OR 'Editorial'/it OR 'Letter'/it OR 'Note'/it OR 'Preprint'/it OR 'Review'/it) | 58 |  |
| #34 | #32 AND ('article'/it OR 'article in press'/it OR 'editorial'/it OR 'letter'/it OR 'note'/it OR 'preprint'/it OR 'review'/it) AND [1990-2024]/py | 58 |  |
| #35 | #32 AND ('article'/it OR 'article in press'/it OR 'editorial'/it OR 'letter'/it OR 'note'/it OR 'preprint'/it OR 'review'/it) AND [1990-2024]/py NOT [animals]/lim | **56** | Final result for SARS-CoV2-specific records |
| #36 | #10 OR #27 | 153 |  |
| #37 | #36 AND ('Article'/it OR 'Article in Press'/it OR 'Editorial'/it OR 'Erratum'/it OR 'Letter'/it OR 'Note'/it OR 'Preprint'/it OR 'Review'/it) | 137 |  |
| #38 | #36 AND ('article'/it OR 'article in press'/it OR 'editorial'/it OR 'erratum'/it OR 'letter'/it OR 'note'/it OR 'preprint'/it OR 'review'/it) AND [1990-2024]/py | 128 |  |
| #39 | #36 AND ('article'/it OR 'article in press'/it OR 'editorial'/it OR 'erratum'/it OR 'letter'/it OR 'note'/it OR 'preprint'/it OR 'review'/it) AND [1990-2024]/py NOT [animals]/lim | **88** | Final result for records on other types of vaccines |
|  |  | **181** | **Total records** |

**Table S2.2:** Search string and results from the **Scopus** database

| *Query* | *Results (n)* | *Remarks* |
| --- | --- | --- |
| ( ( TITLE-ABS ( "influenza*" AND " vaccin*" ) ) AND ( TITLE ( "vaccin*" ) AND TITLE ( "time*" OR "timing*" OR "circadian*" OR "diurnal*" ) AND TITLE ( "immun*" OR "antibod*" OR "respon*" OR "titer*" OR "cellular*" OR "t cell*" OR "humoral*" OR "t-lymphocyt*" ) ) ) AND NOT ( ( ( TITLE-ABS-KEY ( "circadian-rhythm*" ) OR TITLE ( "circadian*" OR "diurnal-variat*" OR "diurnal-rhythm*" OR "chronovaccinat*" OR "chrono-vaccinat*" OR "biological-rhythm*" OR ( "vaccin*" AND "tim*" ) ) ) AND ( TITLE ( "immun*-respons*" OR "humoral-immun*" OR "antibod*-respons*" OR "antibod*-product*" OR "antibody-titer*" OR "vaccin*-effectiv*" OR "vaccin*-efficac*" OR "cellular-immunity" OR "t-lymphocyt*" OR "t-cell*" ) ) ) AND ( TITLE ( "influenza*" AND " vaccin*" ) ) AND PUBYEAR > 1989 AND PUBYEAR < 2026 ) | **70** | Influenza-specific records |
| ( ( TITLE-ABS ( ( "covid*" OR "corona*" OR "SARS-cov*" ) AND "vaccin*" ) ) AND ( TITLE ( "vaccin*" ) AND TITLE ( "time*" OR "timing*" OR "circadian*" OR "diurnal*" ) AND TITLE ( "immun*" OR "antibod*" OR "respon*" OR "titer*" OR "cellular*" OR "t cell*" OR "humoral*" OR "t-lymphocyt*" ) ) ) AND NOT ( ( ( TITLE-ABS-KEY ( "circadian-rhythm*" ) OR TITLE ( "circadian*" OR "diurnal-variat*" OR "diurnal-rhythm*" OR "chronovaccinat*" OR "chrono-vaccinat*" OR "biological-rhythm*" OR ( "vaccin*" AND "tim*" ) ) ) AND ( TITLE ( "immun*-respons*" OR "humoral-immun*" OR "antibod*-respons*" OR "antibod*-product*" OR "antibody-titer*" OR "vaccin*-effectiv*" OR "vaccin*-efficac*" OR "cellular-immunity" OR "t-lymphocyt*" OR "t-cell*" ) ) ) AND ( TITLE ( ( "covid*" OR "corona*" OR "SARS-cov*" ) AND "vaccin*" ) ) ) | **145** | SARS-CoV2-specific records |
| ( ( ( TITLE-ABS ( "vaccin*" ) ) AND ( TITLE ( "vaccin*" ) AND TITLE ( "time*" OR "timing*" OR "circadian*" OR "diurnal*" ) AND TITLE ( "immun*" OR "antibod*" OR "respon*" OR "titer*" OR "cellular*" OR "t cell*" OR "humoral*" OR "t-lymphocyt*" ) ) ) AND NOT ( ( ( ( TITLE-ABS-KEY ( "circadian-rhythm*" ) OR TITLE ( "circadian*" OR "diurnal-variat*" OR "diurnal-rhythm*" OR "chronovaccinat*" OR "chrono-vaccinat*" OR "biological-rhythm*" OR ( "vaccin*" AND "tim*" ) ) ) AND ( TITLE ( "immun*-respons*" OR "humoral-immun*" OR "antibod*-respons*" OR "antibod*-product*" OR "antibody-titer*" OR "vaccin*-effectiv*" OR "vaccin*-efficac*" OR "cellular-immunity" OR "t-lymphocyt*" OR "t-cell*" ) ) ) AND ( TITLE ( "vaccin*" ) ) ) AND NOT ( ( ( TITLE-ABS-KEY ( "circadian-rhythm*" ) OR TITLE ( "circadian*" OR "diurnal-variat*" OR "diurnal-rhythm*" OR "chronovaccinat*" OR "chrono-vaccinat*" OR "biological-rhythm*" OR ( "vaccin*" AND "tim*" ) ) ) AND ( TITLE ( "immun*-respons*" OR "humoral-immun*" OR "antibod*-respons*" OR "antibod*-product*" OR "antibody-titer*" OR "vaccin*-effectiv*" OR "vaccin*-efficac*" OR "cellular-immunity" OR "t-lymphocyt*" OR "t-cell*" ) ) ) AND ( TITLE ( "influenza*" AND " vaccin*" ) ) ) OR ( ( ( TITLE-ABS-KEY ( "circadian-rhythm*" ) OR TITLE ( "circadian*" OR "diurnal-variat*" OR "diurnal-rhythm*" OR "chronovaccinat*" OR "chrono-vaccinat*" OR "biological-rhythm*" OR ( "vaccin*" AND "tim*" ) ) ) AND ( TITLE ( "immun*-respons*" OR "humoral-immun*" OR "antibod*-respons*" OR "antibod*-product*" OR "antibody-titer*" OR "vaccin*-effectiv*" OR "vaccin*-efficac*" OR "cellular-immunity" OR "t-lymphocyt*" OR "t-cell*" ) ) ) AND ( TITLE ( ( "covid*" OR "corona*" OR "SARS-cov*" ) AND "vaccin*" ) ) ) AND PUBYEAR > 1989 AND PUBYEAR < 2026 ) ) AND NOT ( ( ( ( TITLE-ABS ( "influenza*" AND " vaccin*" ) ) AND ( TITLE ( "vaccin*" ) AND TITLE ( "time*" OR "timing*" OR "circadian*" OR "diurnal*" ) AND TITLE ( "immun*" OR "antibod*" OR "respon*" OR "titer*" OR "cellular*" OR "t cell*" OR "humoral*" OR "t-lymphocyt*" ) ) ) AND NOT ( ( ( TITLE-ABS-KEY ( "circadian-rhythm*" ) OR TITLE ( "circadian*" OR "diurnal-variat*" OR "diurnal-rhythm*" OR "chronovaccinat*" OR "chrono-vaccinat*" OR "biological-rhythm*" OR ( "vaccin*" AND "tim*" ) ) ) AND ( TITLE ( "immun*-respons*" OR "humoral-immun*" OR "antibod*-respons*" OR "antibod*-product*" OR "antibody-titer*" OR "vaccin*-effectiv*" OR "vaccin*-efficac*" OR "cellular-immunity" OR "t-lymphocyt*" OR "t-cell*" ) ) ) AND ( TITLE ( "influenza*" AND " vaccin*" ) ) AND PUBYEAR > 1989 AND PUBYEAR < 2026 ) ) AND NOT ( ( ( TITLE-ABS-KEY ( "circadian-rhythm*" ) OR TITLE ( "circadian*" OR "diurnal-variat*" OR "diurnal-rhythm*" OR "chronovaccinat*" OR "chrono-vaccinat*" OR "biological-rhythm*" OR ( "vaccin*" AND "tim*" ) ) ) AND ( TITLE ( "immun*-respons*" OR "humoral-immun*" OR "antibod*-respons*" OR "antibod*-product*" OR "antibody-titer*" OR "vaccin*-effectiv*" OR "vaccin*-efficac*" OR "cellular-immunity" OR "t-lymphocyt*" OR "t-cell*" ) ) ) AND ( TITLE ( "influenza*" AND " vaccin*" ) ) AND PUBYEAR > 1989 AND PUBYEAR < 2026 ) ) AND NOT ( ( ( TITLE-ABS ( ( "covid*" OR "corona*" OR "SARS-cov*" ) AND "vaccin*" ) ) AND ( TITLE ( "vaccin*" ) AND TITLE ( "time*" OR "timing*" OR "circadian*" OR "diurnal*" ) AND TITLE ( "immun*" OR "antibod*" OR "respon*" OR "titer*" OR "cellular*" OR "t cell*" OR "humoral*" OR "t-lymphocyt*" ) ) ) AND NOT ( ( ( TITLE-ABS-KEY ( "circadian-rhythm*" ) OR TITLE ( "circadian*" OR "diurnal-variat*" OR "diurnal-rhythm*" OR "chronovaccinat*" OR "chrono-vaccinat*" OR "biological-rhythm*" OR ( "vaccin*" AND "tim*" ) ) ) AND ( TITLE ( "immun*-respons*" OR "humoral-immun*" OR "antibod*-respons*" OR "antibod*-product*" OR "antibody-titer*" OR "vaccin*-effectiv*" OR "vaccin*-efficac*" OR "cellular-immunity" OR "t-lymphocyt*" OR "t-cell*" ) ) ) AND ( TITLE ( ( "covid*" OR "corona*" OR "SARS-cov*" ) AND "vaccin*" ) ) ) ) | **462** | Records on other types of vaccines |


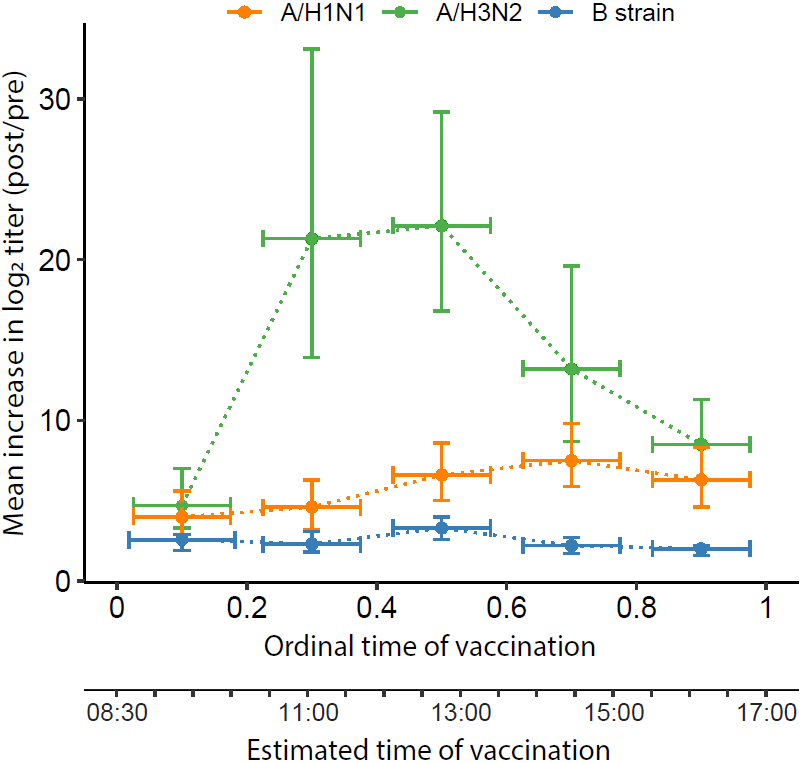

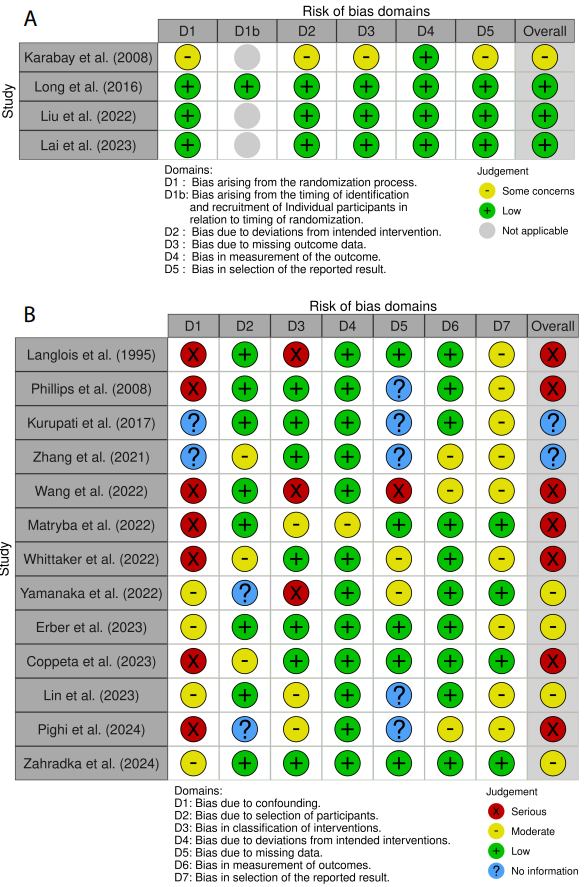


**Figure S3**: Redrawn figure from *Langlois, et al.* (1995) illustrating the difference in the mean rise in log_2_ titer against the three influenza vaccine strains for multiple time groups. Vaccination time was estimated by the order in which the participants were vaccinated during clinic hours (08:30-17:00).

**Figure S4**: Risk of bias in the randomized clinical trials (**A**) and observational studies (**B**) included in the systematic review, assessed using the RoB-2 and ROBINS-I tools, respectively.

180 duplicate records removed before screening

860 records identified:

- Embase + Medline: 181
- Scopus: 677
- Reference list check: 2

**Identification**

680 records screened

656 records excluded based on the title and/or abstract

7 records excluded.

Reason: wrong publication type (n=2), non-human subjects (n=1), or no comparison of vaccine responses between different times of day (n=4).

24 records assessed for eligibility based on a full-text review

**Screening**

13 records excluded.
Reason: observational studies

17 studies included in the systematic literature review

2 trials excluded.
Reason: required data not available (n=1), and vaccine type differed from the other trials (n=1)

**Inclusion**

4 randomized controlled trials

2 trials included in the
meta-analysis

**Figure S5**: The PRISMA (Preferred Reporting Items for Systematic Reviews and Meta-Analyses) flow diagram illustrating the study selection process for the meta-analysis.


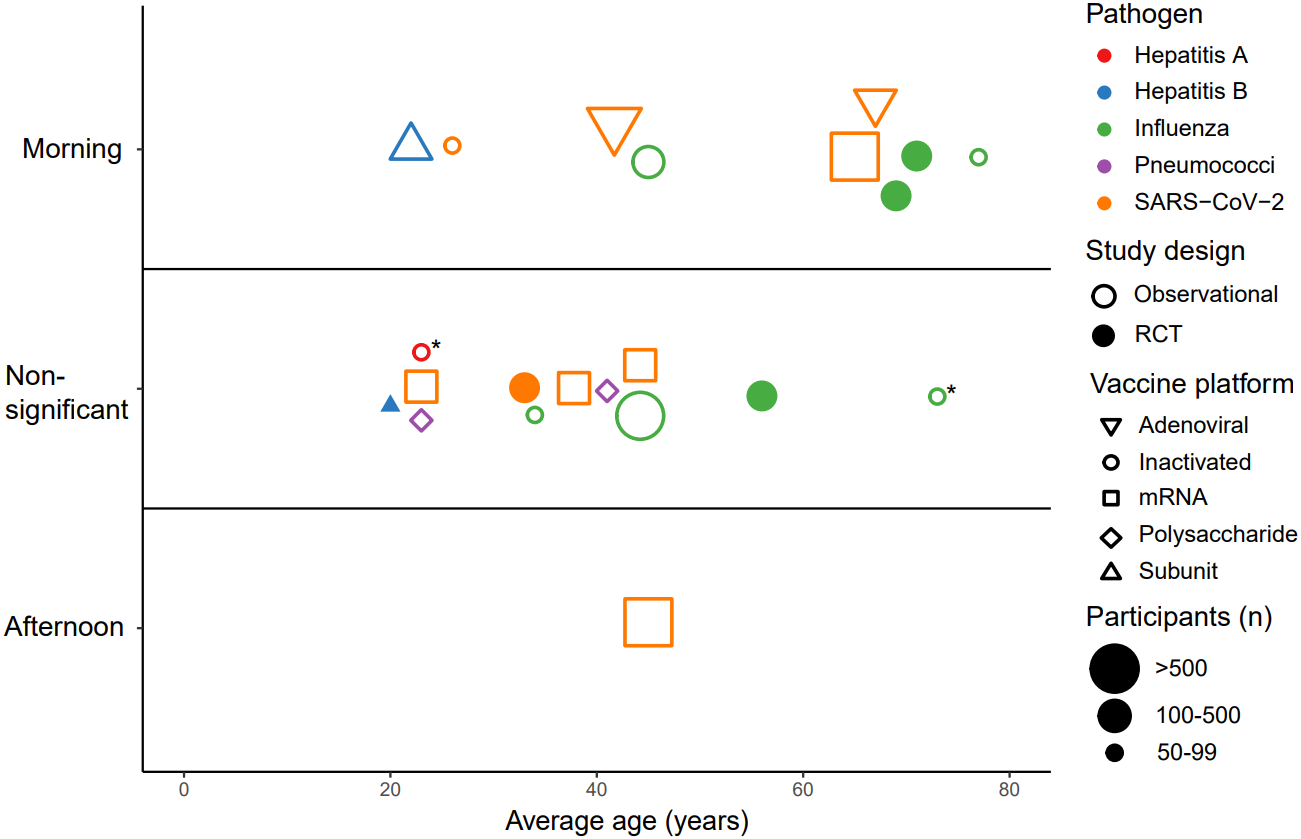


**Figure S6**: The effect of vaccination timing on antibody responses across all age-based subgroups from the included studies. Points represent subgroups that are categorized as “Morning” or “Afternoon” depending on whether the subgroup showed a significantly stronger antibody response for those vaccinated in the morning or afternoon. Subgroups are categorized as “Nonsignificant” if no significant difference (p>0.05) in antibody responses was observed between morning and afternoon vaccination.
* Men vaccinated in the morning showed a significantly stronger antibody response compared to afternoon vaccination.
